# Where Are You From? Finding the Origin of the Recently Observed Sprat in Iceland Using a Panel of SNPs

**DOI:** 10.1002/ece3.72807

**Published:** 2026-03-23

**Authors:** María Quintela, Roger Lille‐Langøy, Christophe Pampoulie, Jón Sólmundsson, Fernando Ayllon, Kevin A. Glover, Florian Berg, Cecilie Kvamme

**Affiliations:** ^1^ Institute of Marine Research Bergen Norway; ^2^ Marine and Freshwater Research Institute Hafnarfjörður Iceland

**Keywords:** ballast water, colonization, European sprat, genetic clustering, SNPs, *Sprattus sprattus*

## Abstract

The European sprat is a small pelagic fish characterized by genetically distinct populations including (a) the Norwegian fjords, (b) the Baltic Sea, (c) an oceanic component ranging from the North Sea, Kattegat–Skagerrak, Celtic Sea and Bay of Biscay, as well as the southern groups such as (d) the Mediterranean (Adriatic) and (e) the Black Sea. Additionally, a self‐recruiting population established in Landvikvannet, a lake on the Norwegian coast of Skagerrak that turned brackish following artificial connection to the sea. Sprat was first reported in Icelandic waters in 2017, and in subsequent years it has become increasingly frequent and has spread along the south and west cost of the country. As the population of origin of this introduction was unknown, we used a panel of 91 SNP loci that display high genetic resolution in this species to characterize the genetic background of 64 sprat individuals collected in Icelandic waters in 2021. Comparison with existing reference data clearly identified the oceanic component as the likely source of the Icelandic sprat, and three potential scenarios can be considered to explain the colonization process. Because adult sprat generally exhibit limited long‐distance movement and are largely sedentary, a scenario involving adult migration into Iceland can be reasonably ruled out. Natural dispersal of eggs or larvae from the Faroe Islands cannot be entirely dismissed under specific conditions, given the complex oceanographic circulation southeast of Iceland. However, an anthropogenically mediated vector—most notably transport via ballast water—remains a more plausible mechanism.

## Graphical abstract

European sprat can be divided into distinct genetic groups in the NE Atlantic, that is, Norwegian fjords, Baltic Sea and an oceanic component. The species has been increasingly reported in Icelandic waters since 2017. To investigate the source of introduction, Icelandic sprat has been genotyped and analyzed in comparison with existing reference data thus concluding that the oceanic component is the most likely source of the introduction.

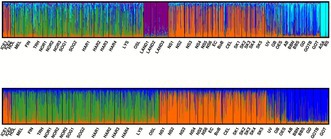

## 1 Introduction

The Arctic Ocean and adjacent seas are unique and vulnerable marine ecosystems facing anthropogenic challenges such as accelerated warming (Adger et al. 2007) – as much as four times the rate of the rest of the world oceans (Rantanen et al. 2022)– and loss of ice cover (Stroeve et al. 2007; Årthun et al. 2019), which promote northward range‐shifts of non‐native species, and are foreseen to increase the liability of these habitats to invasive alien species. The limited functional redundancy of Arctic ecosystems means that the loss of even a single species may lead to pronounced, cascading impacts on the structure and functioning of polar and subpolar ecosystems' (Post et al. 2009).

**FIGURE 1:**
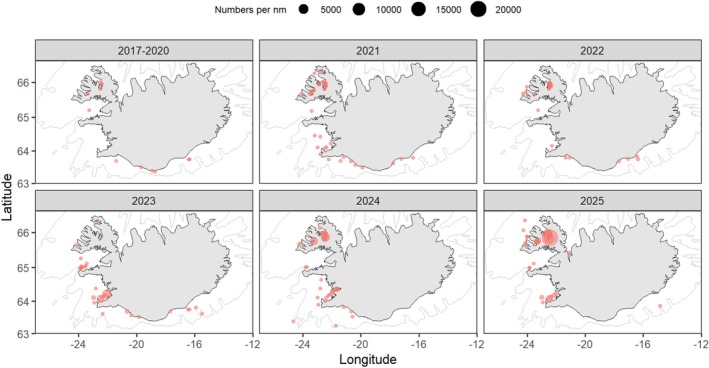
Temporal and spatial distribution of sprat in Iceland. The data mostly originate from annual standardized trawl surveys for groundfish and shrimp, conducted in March and October. The groundfish surveys cover the whole continental shelf, but sprat has not yet been found in the colder waters of NE Iceland.

In Iceland, increased water temperatures, despite rising more moderately than in the Arctic Ocean, have been accompanied by increased abundance and species richness of Atlantic species, especially opportunistic strategists, while species richness for Arctic fish remained similar or decreased (Sólmundsson et al. 2025). In 2021, some 22 non‐native marine taxa including phytoplankton, macroalgae, crustaceans, mollusks, tunicates and fish have been introduced and/or colonized Icelandic waters (ICES 2022). Introduction vectors have been either anthropogenically‐mediated transport or passive transport of plankton or planktonic stages via oceanic currents (see Hoad 2022 for review). Species such as the European flounder *Platichthys flesus* , the brown shrimp *Crangon crangon* and the Atlantic rock crab *Cancer irroratus* , first recorded in 1999, 2003 and 2006 respectively, have rapidly spread and can be already considered invasive (Gíslason et al. 2021; Henke et al. 2025; Thorarinsdóttir et al. 2014). Likewise, the European sprat *Sprattus sprattus* (Linnaeus, 1758) – hereafter referred to as sprat–one of those opportunistic strategists, was first reported in Iceland in 2017 (Figure 1), and subsequent records have been increasingly documented in research trawls with numbers escalating since 2020 (Table S1). Although still uncommon in the southeastern part of Iceland and absent from the east coast, the presence of mature/spent individuals in the southwest and northwest of Iceland confirms that sprat now spawns in Icelandic waters (Hoad 2022; Pálsson et al. 2021).

Sprat is a fast‐growing, small, short‐lived pelagic shoaling fish (Moore et al. 2019; Peck et al. 2012) that plays a crucial ecological role as prey for different piscivorous fishes, marine mammals and seabirds (ICES 2013, 2018). As a batch spawner, it releases pelagic eggs near the surface over a period of up to six months (de Silva 1973). Eggs, together with larvae, are passively advected by horizontal currents thus resulting in high gene flow (Glover et al. 2011; McKeown et al. 2020; Quintela et al. 2020). The species ranges from Morocco to northern Norway, the Baltic Sea, the northern Mediterranean basins (Adriatic Sea) and the Black Sea (Debes et al. 2008). Adult sprat exhibit pronounced seasonal shifts in spatial distribution rather than long‐distance directed migrations typical of larger clupeids such as herring. Sprat are mainly found in shallow areas, such as on the shelf in the North Sea, and only occasionally in deeper waters (Lindegren et al. 2022) like fjords. During the spawning season (spring–summer in the central southern North Sea and Kattegat), the spatial distribution is somewhat more contracted than the autumn‐winter distribution (Lindegren et al. 2022). However, spatial distribution is already influenced by climate change, for example resulting in north and westward shift in the Bay of Biscay and Celtic Sea (Le Luherne et al. 2024).

Throughout most of its natural distribution, sprat sustains multiple fisheries, for many of which the International Council for the Exploration of the Sea (ICES, www.ices.dk) provides management advice. Stock boundaries have been defined based upon Quintela et al. (2020) using a panel of 91 SNPs revealing patterns of differentiation later confirmed by whole genome sequencing (Pettersson et al. 2024). Sprat is divided into several distinct and relatively homogenous genetic groups: (a) the Norwegian fjords, (b) the Baltic Sea, (c) a Northeast Atlantic oceanic component from ranging from the North Sea, Kattegat–Skagerrak, Celtic Sea and the Bay of Biscay, and the southern components composed of (d) the Adriatic and (e) the Black Seas, respectively (Pettersson et al. 2024; Quintela et al. 2020), in addition to Landvikvannet, a once freshwater lake on the Norwegian coast of Skagerrak that became brackish following artificial connection to the sea in 1880 (Quintela et al. 2021). Evidence of genetic admixture, and possibly physical mixing, was also detected in the transition zone between the North Sea and the Baltic Sea, but otherwise, the aforementioned genetic groups are highly genetically distinct.

Here, we aimed to elucidate the genetic origin of the recent invasion of sprat in Icelandic waters by genotyping 64 individuals collected in two different locations in 2021 and comparing them to appropriate reference data sourced from Quintela et al. (2020, 2021).

## 2 Materials and Methods

### 2.1 Sampling and Genotyping

Sprat was sampled in NW Iceland in October 2021 as a bycatch during an annual shrimp survey using a shrimp trawl of 40 mm mesh size in the codend. A total of 64 individuals were collected at two sampling stations located at coordinates 65°57.87 N– 22°32.47 W (*N* = 25 individuals) and 65°48.53 N–22°30.34 W (*N* = 39 individuals), respectively. DNA was extracted from fin clips stored in ethanol using the Qiagen DNeasy 96 Blood & Tissue Kit in 96‐well plates. Individuals were genotyped using the 91 SNP loci published by Quintela et al. (2020). SNP amplification and genotype calling was performed using the Sequenom MassARRAY iPLEX Platform as described by Gabriel et al. (2009).

**FIGURE 2:**
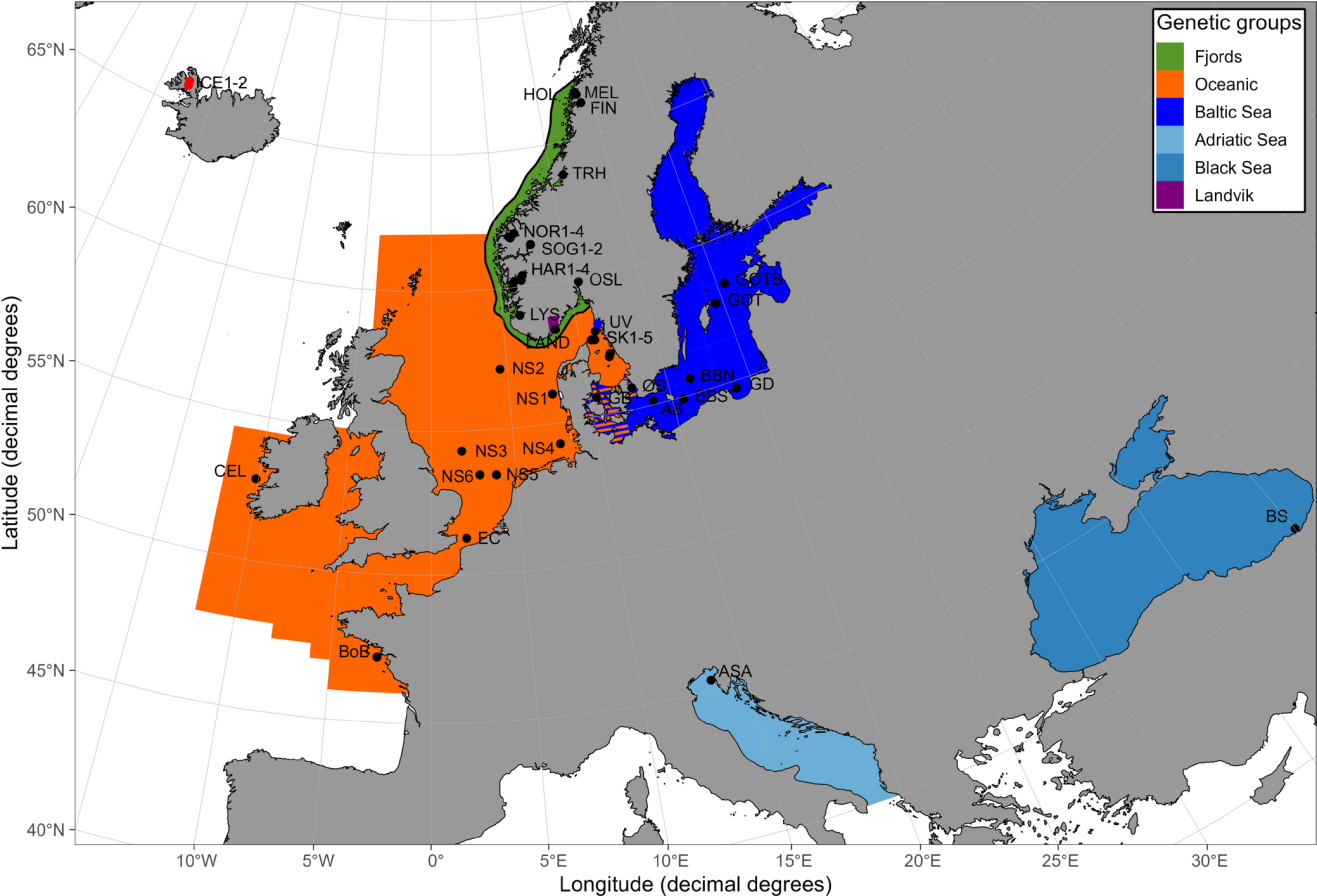
Sampling locations of sprat including two sites in Iceland alongside the reference samples published in Quintela et al. (2020, 2021). Colors depict the genetic clusters known today. The transition zone in Kattegat‐Skagerrak area is depicted by blue and orange bars representing represent the confluence of the oceanic and the Baltic genetic clusters, respectively.

To assess the provenance of the sprat collected in Iceland, the two Icelandic samples were analyzed in combination with a set of 43 reference samples of sprat (total of 2694 individuals) from a range of locations in the Atlantic Ocean as well as the Baltic, Adriatic and Black Seas, all characterized in Quintela et al. (2020) (Figure 2). The reference dataset was completed with individuals from Landvikvannet (Quintela et al. 2021).

### 2.2 Genetic Identification

Statistical analyzes were aimed at identifying the genetic provenance of the Icelandic sprat by analyzing these two samples against the existing reference. Genetic structure was assessed in an unsupervised manner (i.e., without any prior clustering of individuals into groups) with Principal Component Analysis (PCA) using the function *dudi.pca* in the package *ade4* (Dray and Dufour 2007) implemented in the R version 4.5.1 (R Core Team 2025) after replacing missing data with the mean allele frequencies, and using non‐scaled allele frequencies (scale = FALSE). In addition, the Bayesian clustering approach implemented in STRUCTURE v.2.3.4 (Pritchard et al. 2000) was used to identify genetic groups under a model assuming admixture and correlated allele frequencies without using LOCPRIORS (i.e., without prior indication of sample location or potential structure). Ten replicates per K were conducted from *K* = 1 to *K* = 10 with a burn‐in period of 100,000 replications, and a run length of 1,000,000 MCMC iterations using the software ParallelStructure (Besnier and Glover 2013) to reduce computational time.

Supervised genetic structure using geographically explicit samples was assessed through pairwise *F*_ST_ (Weir and Cockerham 1984) computed with Arlequin v.3.5.1.2 (Excoffier et al. 2005). The False Discovery Rate (FDR) correction of Benjamini and Hochberg (1995) was applied to *p*‐values to control for Type I errors. Furthermore, the relationship among samples was examined using Discriminant Analysis of Principal Components (DAPC) (Jombart et al. 2010) implemented in the R package *adegenet* (Jombart 2008) in which groups were defined using geographically explicit locations. To avoid overfitting, both the optimal number of principal components and discriminant functions to be retained were determined using the cross‐validation function (Jombart and Collins 2015; Miller et al. 2020).

## 3 Results

**FIGURE 3:**
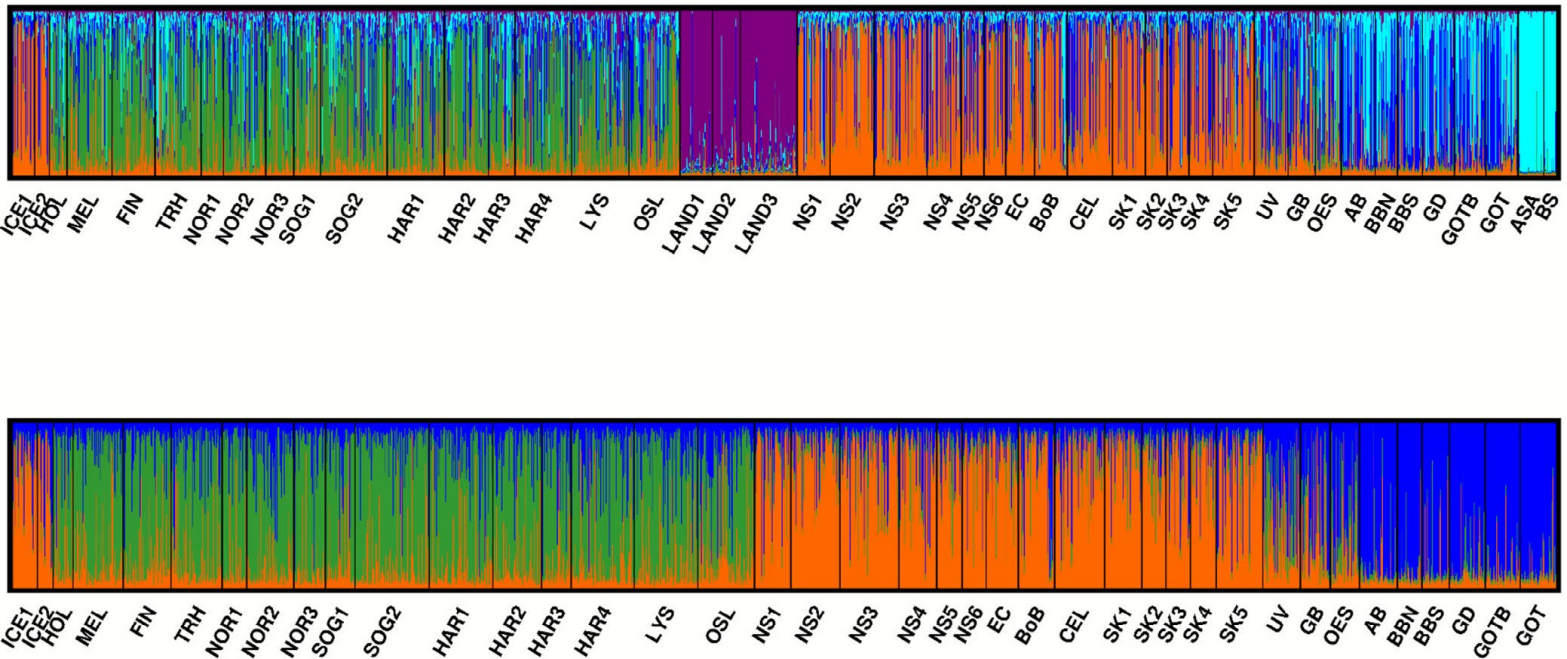
Proportion of individuals' ancestry to clusters after Bayesian cluster analyzes in STRUCTURE assessed from the set of 91 SNP loci to place Icelandic sprat (ICE1, ICE2) in context with the reference samples published in Quintela et al. (2020). The upper barplot depicts the distribution of individuals per cluster at *K* = 5, that is, the most likely number of clusters following Puechmaille's statistics when conducting STRUCTURE with all 45 analyzed samples. Thelower barplot displays the outcome of STRUCTURE at *K* = 3 after trimming the three most unlikely sources of Iceland sprat (i.e., the Adriatic and Black Seas as well as Landvikvannet). Colors depict the three main clusters of sprat in the NE Atlantic: Orange represents the Oceanic cluster, green the Norwegian fjords and dark blue the Baltic Sea. The samples from UV, GB and OES are located in the transition zone between the Atlantic Ocean and Baltic Sea.

The PCA biplot aiming to frame the two Icelandic samples within the 43 reference ones revealed that Landvikvannet was singled out by axis 1, whereas axis 2 separated the Adriatic and Black Seas (Figure S1). The absence of overlap between Icelandic samples and these three geographic locations, together with the results from the DAPC (Figure S2), pairwise *F*_ST_ analyzes (Table S2), and STRUCTURE (Figure 3a, Figures S3 and S4), led to dismiss Landvikvannet, the Adriatic Sea and the Black Sea as potential sources of the Icelandic sprat. Pairwise *F*_ST_ analyzes (Table S2) revealed that Icelandic sprat displayed lower genetic differentiation towards the oceanic cluster (average of 0.022) than to any other, with averages ranging between 0.041 (Iceland vs. Norwegian fjords) and 0.229 (Iceland vs. Black and Adriatic Seas). Furthermore, no maritime routes connect Landvikvannet, the Adriatic Sea, or the Black Sea with Iceland, thus precluding the accidental transport of individuals via ballast water. For all these reasons, Landvikvannet, the Adriatic Sea and the Black Sea were discarded from all analyzes henceforth to gain clarity in the graphic representations. Nonetheless, Icelandic samples were significantly distinct from the remaining oceanic samples (ranging from 0.016–0.041), whereas the oceanic cluster without Iceland showed an average *F*_ST_ of 0.002 (ranging 0.000–0.007).

STRUCTURE analyzes on the trimmed dataset depicted three main units corresponding to the Baltic Sea, coastal sprat (i.e., Norwegian fjords) and oceanic sprat including Iceland, respectively (Figure 3b). The DAPC plot confirmed the clustering of Iceland with the oceanic sprat (Figure S5) while aligning to the pairwise *F*_ST_ outcome as the third axis of the DAPC, accounting for 4.4% of the variation, slightly discriminated the Icelandic samples from the bulk of the oceanic ones.

## 4 Discussion

Sprat was detected in Iceland for the first time in 2017, but its putative source remained elusive. Prior to this observation of sprat in Icelandic waters, genetic approaches covering most of the natural distribution range of the species revealed the presence of several genetic components across the northeast Atlantic, which could be discriminated with a panel of 91 SNPs (Quintela et al. 2020, 2021). During the present study, the recently collected Icelandic samples (2021) were integrated into this existing dataset to examine their potential origin. Results clearly demonstrated a close alignment between Icelandic samples and the oceanic genetic component, that is, the genetic profile displayed across the North Sea, Kattegat–Skagerrak, Celtic Sea and the Bay of Biscay. This finding largely rules out the Norwegian fjords, the Baltic Sea, Landvikvannet and the southern groups such as the Adriatic and Black Sea as potential sources of the introduction/colonization.

A working hypotheses about the introduction vector of the sprat in Icelandic waters is that eggs or larvae could have drifted with ocean currents from spawning grounds such as the Faroe Islands or North Sea (Pálsson et al. 2021). The physical oceanography along the Iceland–Faroe Ridge, characterized by its dominant circulation patterns, does not favor a straightforward westward drift from the Faroes to Iceland in surface or shelf waters, thereby reducing the likelihood of such transport being significant. However this hypothesis remains plausible as the complex oceanic circulation southeast of Iceland suggests that, under specific conditions, the dispersal of sprat eggs and larvae from the Faroe Islands to Iceland cannot be entirely dismissed (Gary et al. 2020; Xu et al. 2015).

A second possible hypothesis for the presence of sprat in Iceland would be the colonization of this region by adults from the oceanic component (North Sea, Celtic Sea or Faroe Islands). Sprat occurs in the Faroes ecoregion (ICES 2023) but has not been yet genetically characterized. In spite of this lack of genetic information, it would not be adventurous to speculate that it could match the oceanic pattern as this has been shown to cover a broad geographic range (McKeown et al. 2020; Quintela et al. 2020). This idea of colonization by adult migration gets support from the fact that the appearance of non‐native species Iceland, such as the pink salmon *Oncorhynchus gorbuscha* (Eliasen and Johannesen 2021), mainly occurred along the Greenland‐Scotland Ridge, with arrival through the Scotland–Faroe Islands mount with subsequent waves of colonization. Also, the Atlantic mackerel *Scomber scombrus* (Astthorsson et al. 2012) migrated along this vector towards Iceland. Similarly, the European flounder, currently classified as invasive (Thorarinsdóttir et al. 2014), was first documented in Iceland in 1999 (Jónsson et al. 2001) and microsatellite analyzes indicated that the Faroese population was its most likely source, thus displacing the hypothesis of introduction via ballast water from the coasts of northwestern Europe (Henke et al. 2025). However, adult sprat usually display limited long‐distance migration and are essentially stationary (Lindegren et al. 2022), thus rendering this hypothesis unlikely.

Thirdly, an introduction driven by an anthropogenic vector cannot be dismissed and seems to be the plausible explanation based on our current knowledge on physical oceanography, and the biological characteristics of the species. In the last three decades, translocation events through released ballast water have been suggested to be the origin of the introduction of several new species that seem to thrive along the Greenland–Scotland Ridge; some of which arrived at larval stage (see Pampoulie et al. 2024 for review). Icelandic waters are now home to species such as the brown shrimp (Jónsdóttir et al. 2016), the Atlantic rock crab (Gíslason et al. 2021; Magnússon et al. 2024), the Newfoundland's razor clam *Ensis terranovensis* (Gunnarsson et al. 2023) and the tunicate *Ciona intestinalis*, first observed in 2003–2019 and likely to have been originally transported via ballast water (Thorarinsdóttir et al. 2014). Recently, the headshield gastropod *Melanochlamys diomedea* was first observed in Iceland, with earlier known distribution confined the Pacific side of North America (de Montety et al. 2024). Maritime transport through ballast water or biofouling are considered as the most likely mode of dispersal to Iceland. The known 10 days' survival of sprat in ballast water further supports the viability of the hypothesis of an anthropogenically‐driven introduction (Wonham et al. 2000 and references therein).

Whereas there is no evidence to reject that Icelandic sprat belongs to the oceanic cluster, the limited genetic differentiation observed among samples within it, both using genetic and genomic tools (McKeown et al. 2020; Pettersson et al. 2024; Quintela et al. 2020), hinders a more precise geographical demarcation of the origin of Icelandic sprat. Significant differentiation was detected between the recently genotyped Icelandic samples and the remaining oceanic ones. The temporal aspect does not seem to be a plausible explanation since reference samples span between 2006 and 2018 whereas the Icelandic samples were collected in 2021. The combined effect of founder effect and drift in the Icelandic samples might however have led to this differentiation. Whereas the sparse set of SNPs used here might not be the best tool to investigate this issue, it must be mentioned that *F*_ST_ per locus in the oceanic cluster became significant for 8% of the markers (see Table S3) when including the Icelandic samples in the analysis, which might provide some indication of genetic drift acting on the less abundant Icelandic population. Likewise, it has been shown that this species was able to colonize and develop a genetically highly distinct population in a brackish lake within few decades (Quintela et al. 2021).

This study provides the first genetic evidence that sprat occurring in Iceland belong to the oceanic cluster spanning the North Sea, Kattegat‐Skagerrak, the Celtic Sea, and the Bay of Biscay. The detection of significant, though subtle, genetic differentiation between Icelandic and the remaining oceanic samples suggests that founder effects and genetic drift may already be shaping the Icelandic population. Furthermore, recent colonization events leading to substantial population divergence over short timescales have been reported for this species (Quintela et al. 2021).

Further genomic work will be essential to unravel the colonization pathways and connectivity of sprat across the North Atlantic. High‐resolution genomic data, including full reference genomes and the characterization of putative structural variants such as inversions, could clarify fine‐scale population structure and help trace the specific origin of the Icelandic population (Thorburn et al. 2023). Moreover, the inclusion of samples from uncharacterized regions such as the Faroe Islands will be key to testing the hypothesis of colonization via the Greenland–Scotland Ridge. Together, such efforts will refine our understanding of how oceanographic processes and climate‐driven range expansions shape connectivity and population differentiation in pelagic fishes of the North Atlantic.

## Author Contributions

**María Quintela:** conceptualization (equal), formal analysis (lead), investigation (equal), methodology (equal), visualization (supporting), writing – original draft (lead). **Roger Lille‐Langøy:** data curation (lead), methodology (lead), writing – review and editing (supporting). **Christophe Pampoulie:** conceptualization (equal), data curation (equal), writing – original draft (supporting), writing – review and editing (supporting). **Jón Sólmundsson:** conceptualization (equal), data curation (equal), visualization (equal), writing – original draft (supporting), writing – review and editing (equal). **Fernando Ayllon:** data curation (supporting), methodology (equal), validation (lead), writing – review and editing (equal). **Kevin A. Glover:** conceptualization (equal), project administration (supporting), supervision (equal), writing – review and editing (equal). **Florian Berg:** conceptualization (equal), project administration (equal), resources (supporting), visualization (equal), writing – review and editing (equal). **Cecilie Kvamme:** conceptualization (equal), funding acquisition (lead), project administration (lead), resources (lead), writing – review and editing (supporting).

## Funding

This work was supported by Norwegian Department of Trade and Fisheries.

## Disclosure

Authors' Statements: Authors contributed to the text, agreed with its content and approved it for submission. Sprat is a commercial species that was collected in scientific surveys where all research met the ethical guidelines of the study countries.

## Conflicts of Interest

The authors declare no conflicts of interest.

## Supporting information


**Data S1:** ece372807‐sup‐0001‐Supinfo.zip.

## Data Availability

The genotype raw data of the Icelandic sprat used in this study can be publicly accessed from the electronic archive of the Institute of Marine Research at https://hdl.handle.net/11250/3212171.
