# Supplementary material for: Where Are You From? Finding the Origin of the Recently Observed Sprat in Iceland Using a Panel of SNPs
Source: Ecol Evol. 2026 Mar 23;16(3):e72807. doi: 10.1002/ece3.72807 (PMC13093719; doi:10.1002/ece3.72807)
Supplement: Supplementary file 1 — Data S1: ece372807‐sup‐0001‐Supinfo.zip. [file ECE3-16-e72807-s001.zip › ECE-2025-08-02220.R1__Supplement.docx]

**Where are you from? Finding the origin of the recently observed sprat in Iceland using a panel of SNPs**

**FIGURES**

**
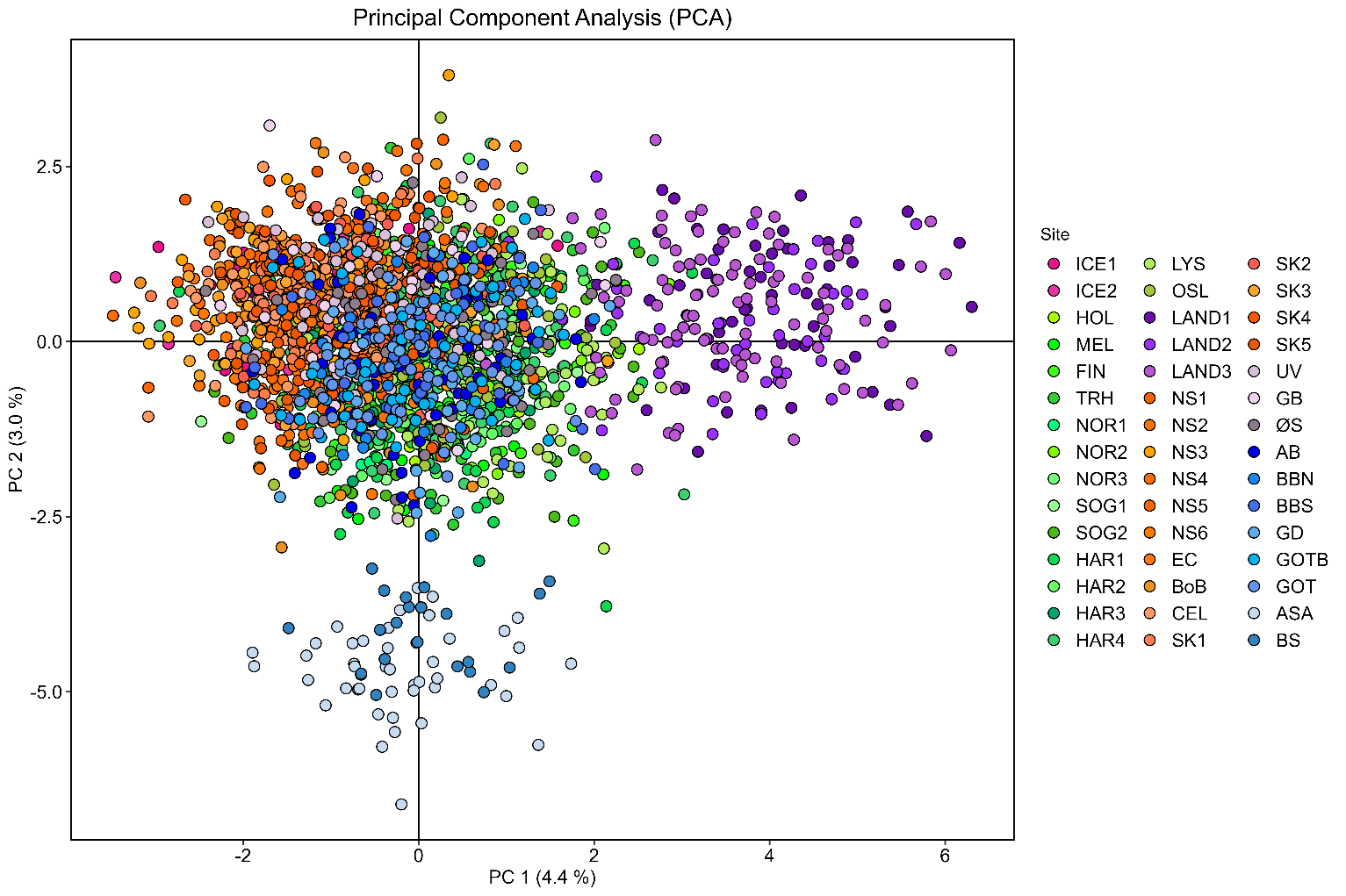
**

**Fig. S1.** Principal Component Analysis biplot of the Icelandic samples (ICE 1, ICE 2) combined with the 43 reference samples genotyped at 91 SNP loci described in Quintela et al. (2020). Individuals from different sampling sites are represented by coloured dots, and detailed information regarding sampling coordinates and sampling year can be found in Quintela et al. (2020, 2021).

| a)  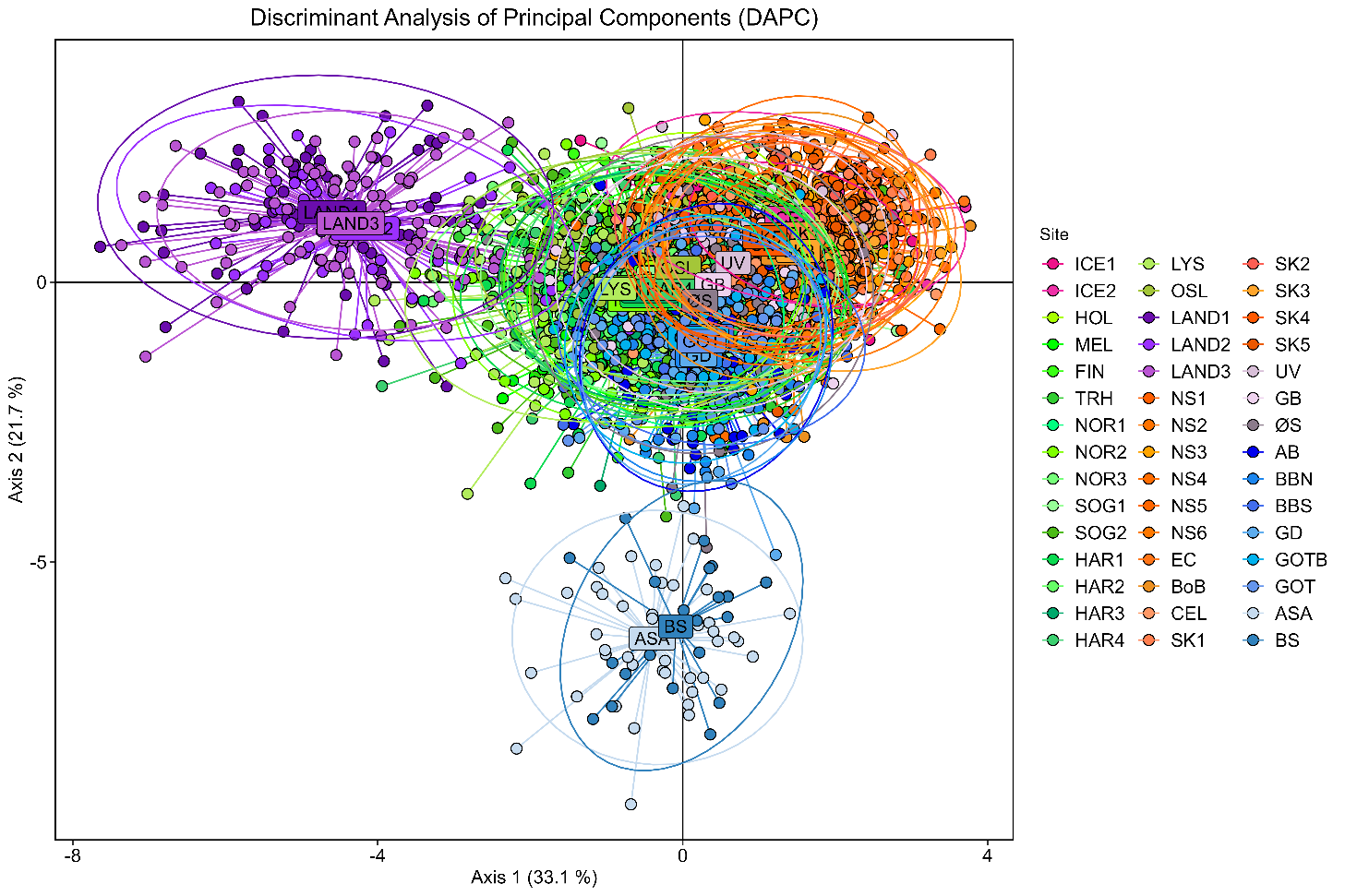 |
| --- |
| b)  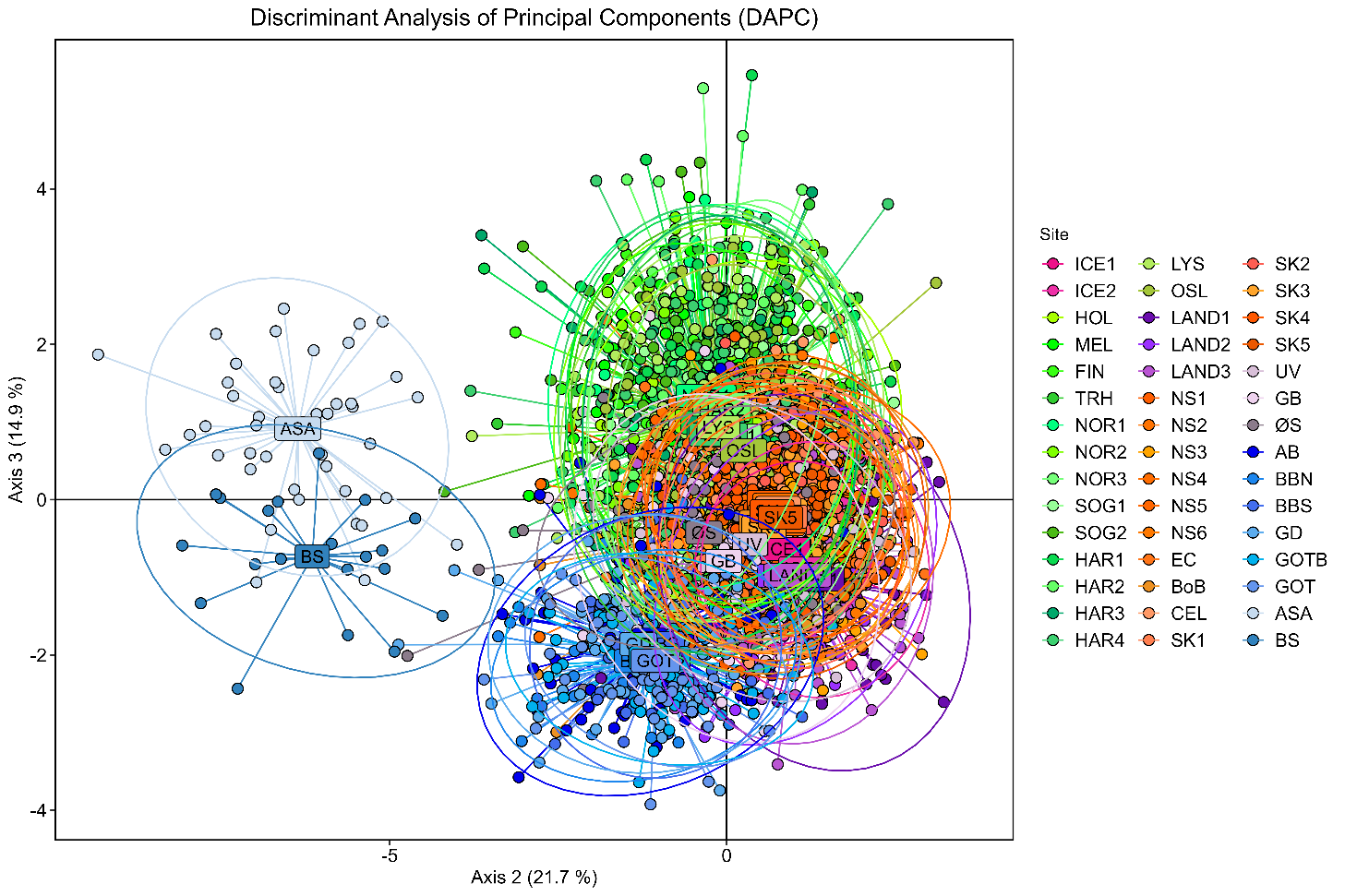 |

| c)  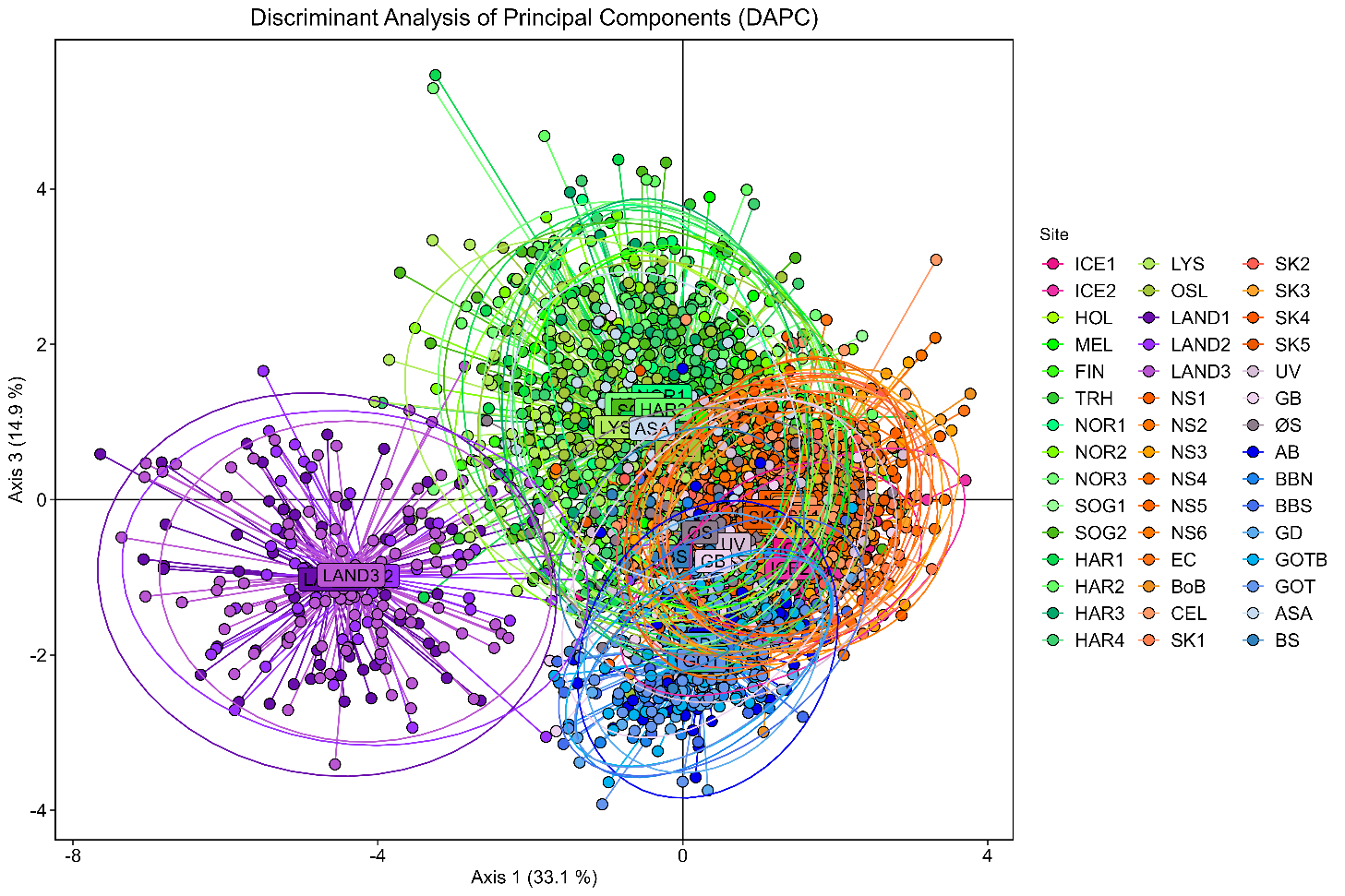 |
| --- |

**Fig. S2.** Genetic differentiation among sprat samples genotyped with 91 SNP loci using Discriminant Analysis of Principal Components (DAPC) after retaining 60 principal components and 3 discriminant functions: a) axis 1 and 2, b) axis 2 and 3, and c) axis 1 and 3. Individuals from different sampling sites are represented by coloured dots and detailed information regarding sampling coordinates and sampling year can be found in Quintela et al. (2020, 2021).

**Fig. S3.** A posteriori analysis of the STRUCTURE outcome for the set of 45 samples genotyped at 91 SNP loci following Puechmaille and Evanno’s methods. Puechmaille’s statistics −developed to account for sampling unevenness and improve the detection of the correct population structure− favoured the solution of K=5 clusters followed by K=6. In contrast, Evanno’s method pointed at K=2, followed by K=4. Evanno’s test showed the largest support for K=2, which separates the Norwegian fjords plus Landvik (population that most likely was originated from fjord sprat that got established in the lake when it was connected to the sea) from the oceanic and Baltic sprat. But this solution could also be a product of the so-called “The K = 2 conundrum” (Janes et al., 2017) as it has been shown that, with a representative number of markers, ΔK frequently identifies K = 2 as the top level of hierarchical structure, even when more subpopulations are present. However, the second-best supported solution for Evanno’s test is K=4, which identifies the Norwegian fjords, Landvik, the oceanic cluster, and a third component of brackish samples (Baltic and Black Sea) together with the Adriatic Sea. The barplots corresponding to all the solutions mentioned can be found in **Fig. S4**.

Janes, J. K., Miller, J. M., Dupuis, J. R., Malenfant, R. M., Gorrell, J. C., Cullingham, C. I., and Andrew, R. L. (2017). The K = 2 conundrum. Molecular Ecology, 26(14), 3594-3602. doi: <https://doi.org/10.1111/mec.14187>

| **K2**  **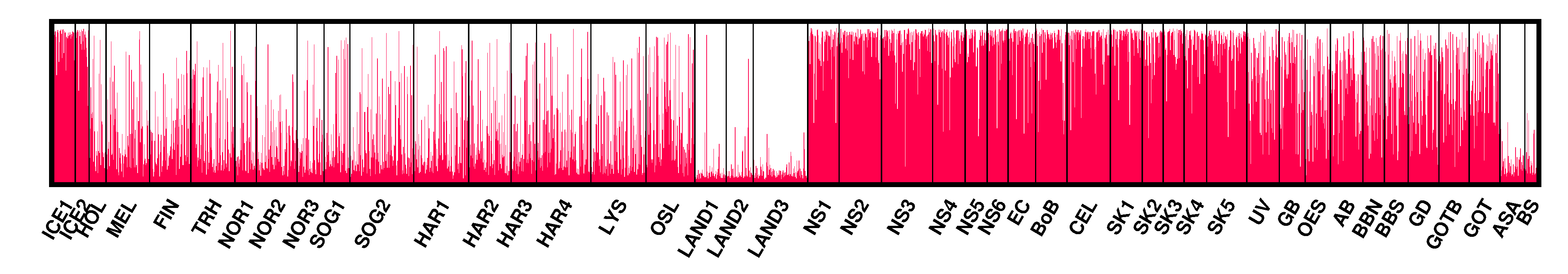** |
| --- |
| **K4**  **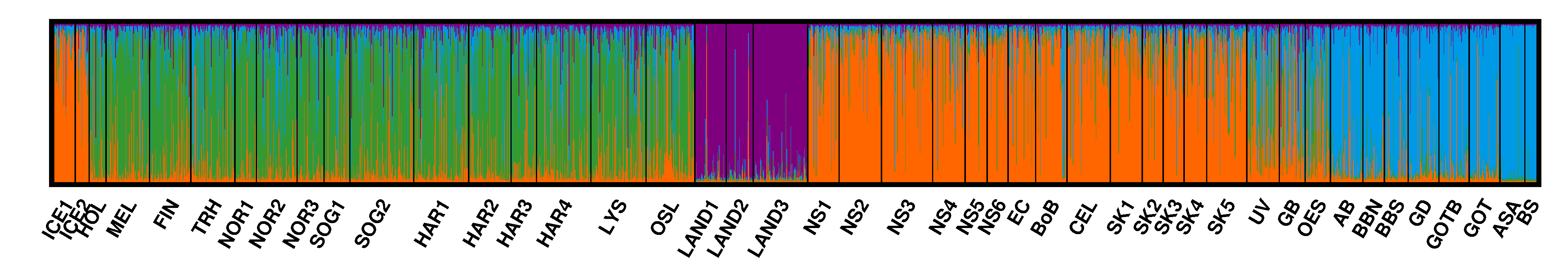** |

| **K5**  **** |
| --- |
| **K6**  **** |

**Fig. S4.** Proportion of individuals’ ancestry to cluster at K = 2, 4, 5 and 6 after Bayesian cluster analyses performed in STRUCTURE and assessed for the total 45 samples genotyped at 91 SNP loci. “Hybrid Z” depicts the transition zone located in Kattegat-Skagerrak area where the North Sea and the Baltic Sea meet. ASA and BS account for the Adriatic Sea and Black Sea, respectively.

| a)  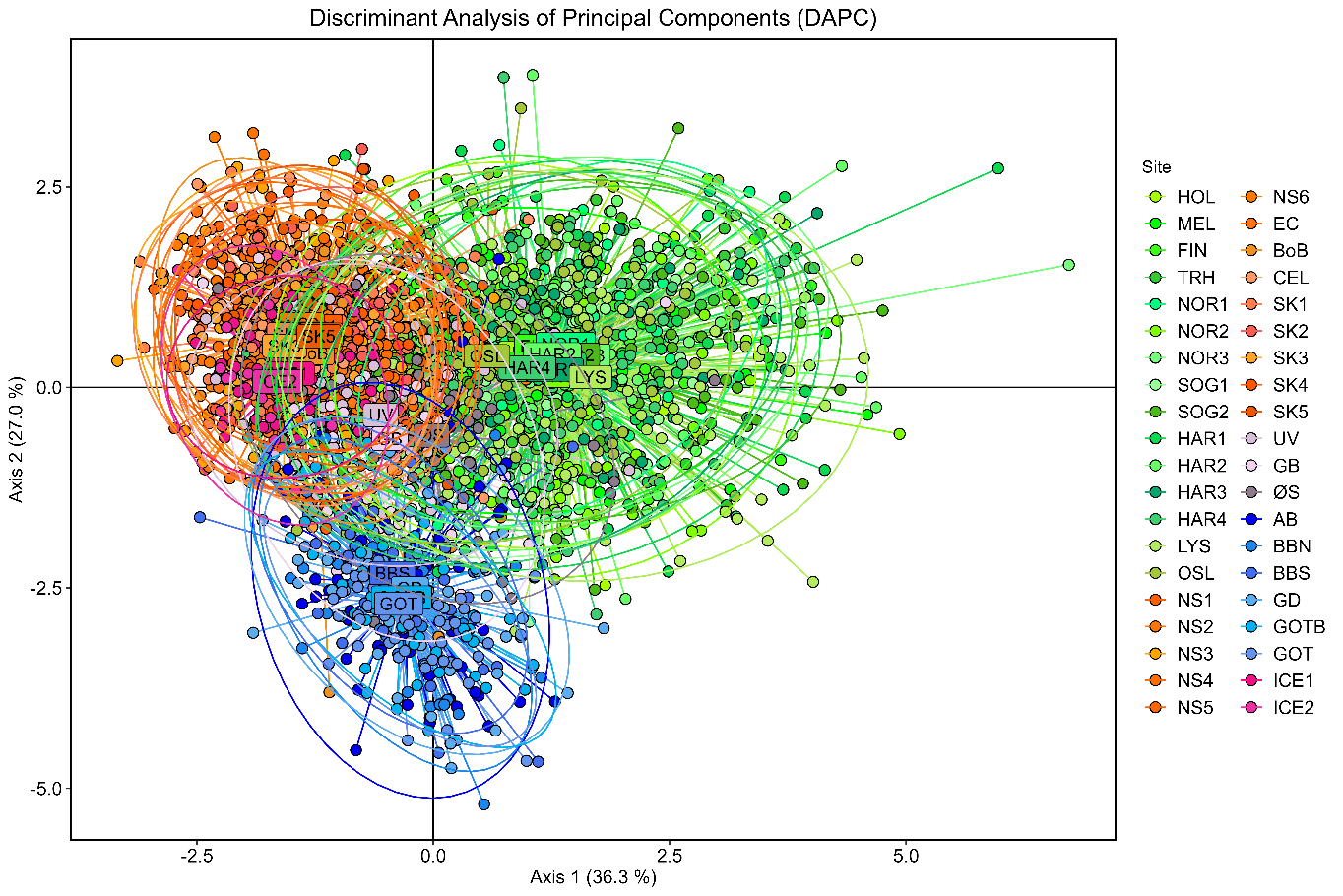 |
| --- |
| b)  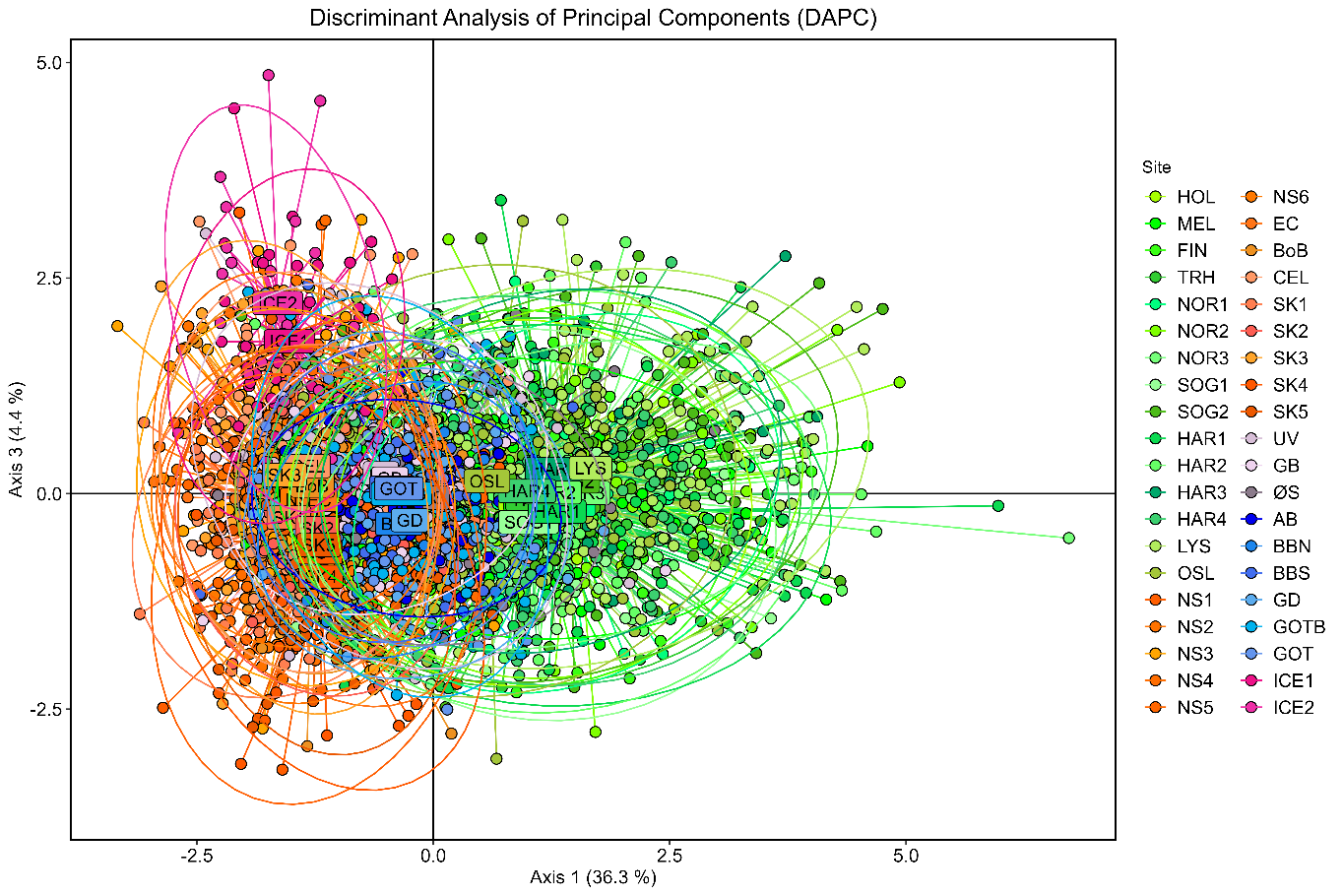 |

**Fig. S5.** Discriminant Analysis of Principal Components (DAPC) along axis 1 and 2 (a) and 1 and 3 (b) of sprat collected in the Norwegian fjords, Baltic Sea and oceanic locations in the Atlantic, and genotyped with 91 SNP loci. DAPC was conducted after retaining 60 principal components and 3 discriminant functions. Colours depict different sampling sites.

**TABLES**

**Table S1.** Number of individual sprat observed in Iceland and number of samples. * Data for 2025 was retrieved on the 5^th^ November 2025.

| **Year** | **No individuals** | **No samples** |
| --- | --- | --- |
| 2017 | 1 | 1 |
| 2019 | 3 | 2 |
| 2020 | 36 | 8 |
| 2021 | 684 | 37 |
| 2022 | 1340 | 23 |
| 2023 | 26473 | 30 |
| 2024 | 11821 | 35 |
| 2025* | 62531 | 32 |

**Table S2.** Pairwise F_ST_ (excel document)

**Table S3.** Major allele frequency of the loci that displayed significant genetic differentiation in the oceanic cluster after including the Icelandic samples. Heatmap should be read per locus across samples with red colours depicting the largest values and green colours the lowest ones.

| **Locus** | **Ssp202** | **Ssp210** | **Ssp220** | **Ssp229** | **Ssp275** | **Ssp276** | **Ssp297** |
| --- | --- | --- | --- | --- | --- | --- | --- |
| **ICE1** | 0.676 | 0.949 | 0.513 | 0.778 | 0.936 | 0.910 | 0.500 |
| **ICE2** | 0.417 | 0.920 | 0.396 | 0.696 | 0.940 | 0.960 | 0.500 |
| **NS1** | 0.361 | 0.636 | 0.698 | 0.620 | 0.991 | 0.947 | 0.518 |
| **NS2** | 0.493 | 0.562 | 0.647 | 0.572 | 0.994 | 0.896 | 0.592 |
| **NS3** | 0.416 | 0.618 | 0.571 | 0.583 | 0.995 | 0.876 | 0.574 |
| **NS4** | 0.430 | 0.613 | 0.640 | 0.519 | 1.000 | 0.915 | 0.570 |
| **NS5** | 0.450 | 0.638 | 0.603 | 0.649 | 0.988 | 0.913 | 0.383 |
| **NS6** | 0.579 | 0.667 | 0.581 | 0.658 | 1.000 | 0.882 | 0.541 |
| **EC** | 0.510 | 0.677 | 0.542 | 0.617 | 0.990 | 0.860 | 0.612 |
| **BoB** | 0.439 | 0.670 | 0.600 | 0.583 | 1.000 | 0.891 | 0.480 |
| **CEL** | 0.538 | 0.597 | 0.638 | 0.514 | 0.993 | 0.926 | 0.547 |
| **SK1** | 0.519 | 0.500 | 0.679 | 0.621 | 1.000 | 0.871 | 0.632 |
| **SK2** | 0.471 | 0.618 | 0.649 | 0.569 | 1.000 | 0.921 | 0.446 |
| **SK3** | 0.364 | 0.694 | 0.694 | 0.736 | 1.000 | 0.875 | 0.408 |
| **SK4** | 0.525 | 0.613 | 0.788 | 0.650 | 1.000 | 0.890 | 0.608 |
| **SK5** | 0.430 | 0.603 | 0.600 | 0.562 | 0.993 | 0.897 | 0.552 |
